# Study on disaster-causing probability evaluation of gas pipeline in karst area

**DOI:** 10.1371/journal.pone.0316820

**Published:** 2025-02-03

**Authors:** Qiaochu Li, Peng Zhang

**Affiliations:** 1 School of Economics and Management, Southwest Petroleum University, Chengdu, China; 2 School of Civil Engineering and Geomatics, Southwest Petroleum University, Chengdu, China; National Cheng Kung University, TAIWAN

## Abstract

In this paper, a disaster-causing probability evaluation method of gas pipelines in karst areas based on disaster system theory and vulnerability theory is proposed. The hazard evaluation index system of gas pipeline disaster events in karst areas is established from three aspects: the activity of disaster-prone environment, the risk of disaster factors and the vulnerability of disaster-bearing bodies. Combined with the advantages of information transmission and updating of the Bayesian network model, the hazard probability of disaster events is gradually calculated from a multi-level perspective. Based on the structural reliability theory, the vulnerability evaluation function of gas pipelines in karst areas is established by considering the interaction between disaster hazard intensity and disaster resistance ability. Meanwhile, the design checkpoint method and finite element simulation are used to calculate the vulnerability probability. Finally, a new disaster-causing probability evaluation approach for gas pipelines in karst areas is formed, which comprehensively considers the event hazard and the pipeline vulnerability. The work presented in this paper can provide a reference for the safety management and accident prevention of gas pipelines crossing the karst areas.

## 1. Introduction

China’s goals of carbon peaking and carbon neutrality have clarified the strategic direction and requirements for comprehensive green transformation [1]. As a clean, efficient and high-quality fossil energy, natural gas has become an important transitional energy source for the third energy transition, which drives the continuous construction of gas pipeline network. The karst landforms in China are widely distributed, concentrated in the southwest carbonate geological areas such as Sichuan, Guizhou, Yunnan, Hunan, Guangxi, and Hubei. The southwest China is rich in natural gas resources, and its underground gas pipeline network is widely distributed. In this regard, the gas pipelines inevitably pass through the karst areas, and the geological development poses significant hidden dangers to the safe and stable operation of buried pipelines [2]. Meanwhile, the natural environment in southwest China is complex and fragile. The terrain is mainly mountainous and plateau, with broken surface rocks and rainy climate. In addition, the gas pipeline disaster in karst areas is a special case of disaster. Specifically, it is dominated by natural factors, but coupled with human factors to form a more complex system structure. In this regard, karst collapse is easy to form under the coupling influence of natural events such as rainstorm and human activities such as third-party construction. It will lead to the failure and rupture of buried gas pipelines, and further induces secondary disasters such as fires and explosions. Those disasters have a negative impact on energy supply, social stability and economic development, and seriously affect the high-quality transformation of China’s energy industry [3]. Therefore, it is necessary to scientifically and accurately evaluate the disaster-causing probability of gas pipelines in karst areas, and provide theoretical guidance for disaster emergency response and rescue work.

The evaluation of disaster-causing possibility includes failure analysis and probability calculation [4]. In recent years, pipeline disasters caused by soil collapse have occurred frequently. Scholars have begun to pay attention to the failure analysis of buried pipelines in soil collapse areas, and computer numerical simulation methods with advantages such as high accuracy and strong realism have been widely used [5]. The numerical simulation method sets model parameters and boundary conditions based on the actual engineering characteristics, and further establishes a three-dimensional finite element calculation model by combining intelligence analysis software such as ABAQUS and ADINA. In this regard, the mechanical response of pipelines under different collapse scenarios and different operating conditions can be simulated, so as to accurately investigate the failure characteristics and disaster-causing laws of buried pipelines in soil collapse areas [6]. Trifonov [7] used the finite element analysis method to evaluate the stress-strain response of the cross-fault pipelines in the state of uniform internal pressure based on two different types of fault representations. Ukan et al. [8] studied the mechanical response of cross-fault pipelines with different diameter-thickness ratios and steel grades. Naeen et al. [9] investigated the deformational behavior of buried pipelines under soil collapse by applying 3D continuum finite element modeling. With the development of disaster probability-causing evolution for pipelines in soil collapse areas, in addition to technical analysis of pipeline failure, attention has been paid to the calculation of disaster-causing probability combined with multiple factors such as society, economy, and environment [10]. The commonly used models include fault tree [11], Bowtie node [12] and Bayesian network [13]. Meanwhile, on the basis of system structure analysis, the analytic methods such as cluster analysis, analytic hierarchy process, fuzzy analysis and system analysis are further used to carry out probability quantitative calculation [14]. Phan et al. [15] established a strain failure probability calculation model for buried pipelines crossing strike-slip faults based on the artificial neural network method. However, the predicted strain may be significantly different from the actual strain, thus significantly affecting the accuracy of possibility evolution. Aslkhalili et al. [16] established the calculation method of failure probability of buried pipelines under the influence of large lateral soil displacements based on the first-order reliability method, and used Python programming software to develop calculation code. Alvarado-Franco et al. [17] established the failure probability analysis model of buried pipelines under the influence of landslide based on the Monte Carlo simulation method, and conducted a case study using a pipeline in central Colombia. Zheng et al. [18] established the failure probability calculation model of buried pipelines under the influence of permanent ground displacement based on the finite difference method. However, the strain capacity was determined using existing equations in the literature. Compared with the numerical simulation results, its accuracy is insufficient.

The above studies provides an effective reference for the disaster-causing probability evaluation of gas pipelines in soil collapse areas, but they still have limitations in the following aspects: firstly, the existing studies mainly focus on the mechanical failure process of pipelines under the influence of soil displacement, and conducts quantitative analysis based on engineering parameters such as collapse size, pipeline operation characteristics, and site soil properties [9, 19, 20]. The comprehensive analysis combined with disaster system theory is relatively lacking, and multiple factors such as society, economy and environment are not comprehensively considered. Therefore, it is often difficult to achieve accurate measurement of disaster-causing probability. Secondly, most of the existing studies only focus on the hazard analysis of disaster events on buried pipelines, without considering the pipeline vulnerability [21–23]. In this regard, the important influence of pipeline anti-external load ability on disaster-causing process is ignored. In addition, the collapse scenario mainly focuses on earthquake collapse, landslide, mining collapse, etc. It is worth noting that karst collapse has the characteristics of hiddenness and suddenness, and once it happens, it will have a devastating impact on the pipeline system. Therefore, the targeted research on it needs to be further strengthened.

In this regard, this study proposes a method to analyze the disaster-causing probability of gas pipelines in karst areas based on disaster system theory and vulnerability theory. On the one hand, the hazard evaluation index system of gas pipeline disaster events in karst areas is established from multiple dimensions including the activity of disaster-prone environment, the risk of disaster factors and the vulnerability of disaster-bearing bodies. Combined with the advantages of information transmission and updating of the Bayesian network model, the hazard probability of disaster events is gradually calculated from a multi-level perspective. On the other hand, considering the pipeline vulnerability level and its relationship with the disaster type and intensity, pipeline structure and function, disaster and pipeline space-time configuration, etc., a mathematical model system is established based on the finite element simulation results and polyethylene (PE) pipeline characteristics. Combined with the design checkpoint method, the vulnerability probability of gas pipelines is calculated from the perspective of the interaction between disaster hazard intensity and pipeline resistance capacity. Finally, a new disaster-causing probability evaluation approach for gas pipelines in karst areas is formed, which comprehensively considers the event hazard and the pipeline vulnerability. It is helpful to expand the research ideas in the field of risk assessment of gas pipeline disasters, and provide theoretical basis for scientific, accurate and comprehensive investigation of disaster-causing probability of gas pipelines in karst sinkhole prone areas.

## 2. Methods

### 2.1. Hazard analysis of disaster events

The hazard probability of disaster events is defined as the occurrence probability of all types of disaster events that threaten the safety of human beings and material wealth in the area. That is, the probability distribution of multiple risk inducing factors turning into serious disaster events [24].

#### 2.1.1. Establishment of hazard evaluation index system

Based on the disaster system theory, the hazard evaluation index system is established from three aspects: the activity of disaster-prone environment, the risk of disaster factors and the vulnerability of disaster-bearing bodies.

**Fig 1:**
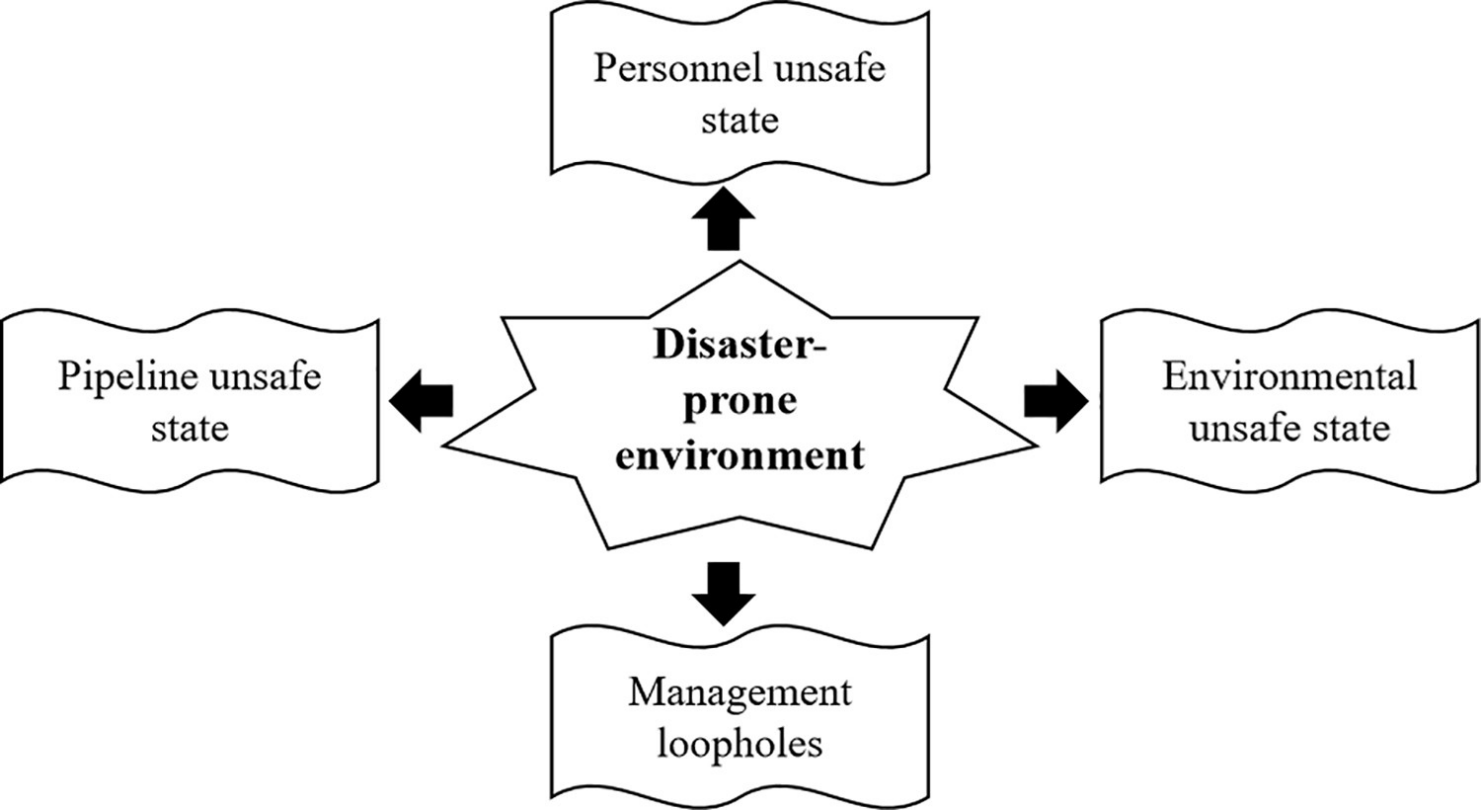
The structure of disaster-prone environment.

*(1) The disaster-prone environment*. In a broad sense, the disaster-prone environment refers to a comprehensive social-natural system composed of multiple factors of atmosphere, lithosphere, biosphere, hydrosphere, and social material and cultural sphere. However, the formation of the disaster-prone environment is not simply the direct superposition of the related factors, but depends on the material circulation, energy flow and information transmission among various factors. The disaster-prone environment of gas pipeline disasters in karst areas is the breeding place of disaster factors, and it is also the indirect medium of the interaction between disaster factors and disaster-bearing bodies. The activity of disaster-prone environment (*a*) is closely related to the personnel unsafe state (*k*_1_), pipeline unsafe state (*k*_2_), environmental unsafe state (*k*_3_) and management loopholes (*k*_4_), as shown in Fig 1.

Specifically, the personnel unsafe state (*k*_1_) mainly includes the low level of education (*t*_1_), the lack of professional skills (*t*_2_), the weak awareness of pipeline protection (*t*_3_), weak safety awareness (*t*_4_) and insufficient emergency response capacity (*t*_5_). The pipeline unsafe state (*k*_2_) mainly includes the pipeline aging (*t*_6_), pipeline corrosion (*t*_7_), the failure of safety facilities (*t*_8_) and construction technology defect (*t*_9_). The environmental unsafe state (*k*_3_) mainly includes the intensity of human activity (*t*_10_), construction and vibration (*t*_11_), groundwater extraction (*t*_12_), economic development level (*t*_13_), legal environment (*t*_14_), karst geological development (*t*_15_), groundwater activity (*t*_16_), overburden characteristics (*t*_17_), structural condition (*t*_18_), topographic features (*t*_19_) and meteorological condition (*t*_20_). The management loopholes (*k*_4_) mainly includes the lack of safety supervision (*t*_21_), the lack of safety publicity (*t*_22_), inadequate emergency support (*t*_23_) and unreasonable rules and regulations (*t*_24_).

**Fig 2:**
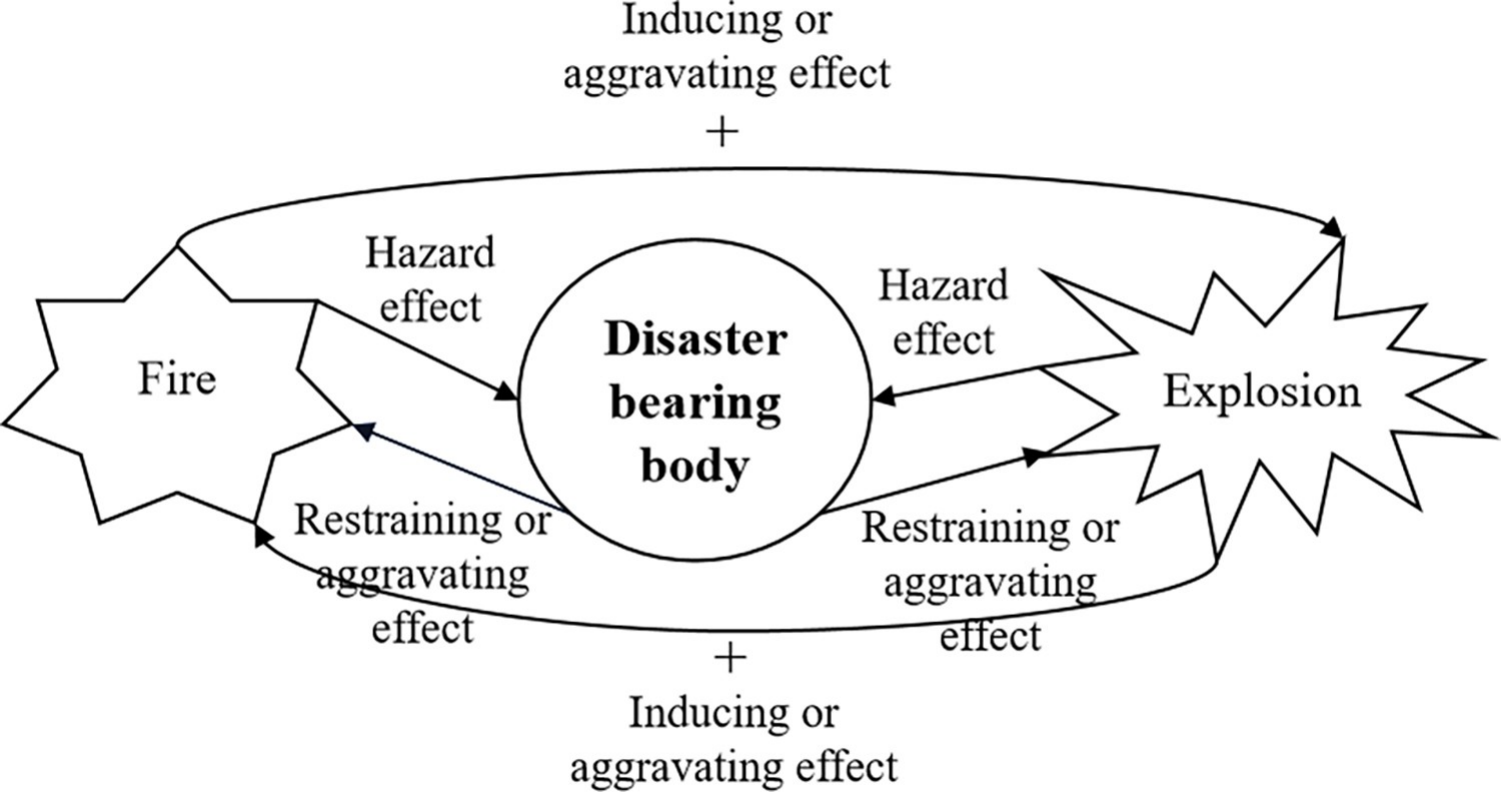
The structure of disaster factors.

*(2) The disaster factors*. The disaster factors are the driving force and direct cause of hazards, which are manifested as various sudden extreme events in natural or social environments that threaten human life, cause material property losses, and cause ecological damage. They include both natural anomalies such as mudslides, tsunamis, tornadoes, and earthquakes, as well as anthropogenic anomalies such as desertification, hazardous spills, traffic accidents, and explosions. The disaster factors of gas pipelines in karst areas are the direct causes of casualties, economic losses and environmental damage, mainly including fire thermal radiation and explosion shock wave overpressure. The risk of disaster factors is mainly influenced by the probability, intensity, scope and duration of fire and explosion hazards. Among the various disaster factors caused by gas pipeline failure and leakage, the occurrence of a single disaster can lead to the subsequent occurrence of other secondary disasters. Therefore, the disaster factors of gas pipelines in karst areas show a chain formation process. The risk of disaster factors (*b*) is closely related to the fire risk (*k*_5_) and explosion risk (*k*_6_), as shown in Fig 2.

Specifically, the fire risk (*k*_6_) mainly includes the source of fire (*t*_25_), thermal radiation flux (*t*_26_), smoke and dust (*t*_27_), noxious fumes (*t*_28_), the types of gas leakage (*t*_29_), the scope of fire impact (*t*_30_), radiation time (*t*_31_), distance (*t*_32_) and space-limited situation (*t*_36_). The explosion risk (*k*_5_) mainly includes the shock wave overpressure (*t*_33_), noise (*t*_34_), the source of fire (*t*_25_), the types of gas leakage (*t*_29_), distance (*t*_32_), the scope of explosion impact (*t*_35_) and space-limited situation (*t*_36_).

**Fig 3:**
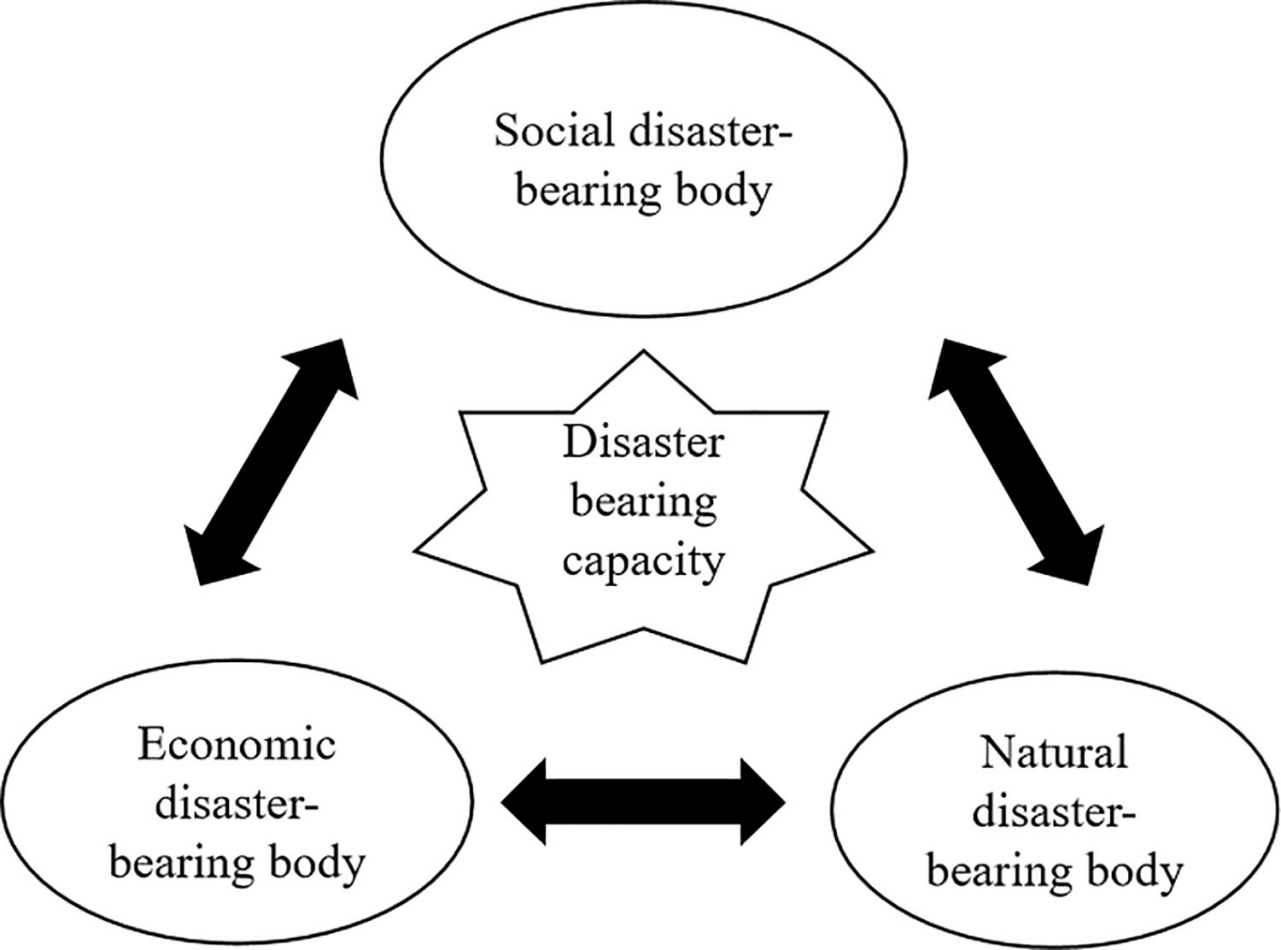
The structure of disaster-bearing bodies.

*(3) The disaster-bearing body*. The disaster-bearing bodies refers to the direct natural and social subjects affected by disasters, including various social resources such as human life, material property, social production and operation systems, as well as various natural resources such as animals, plants, soil, water sources, and atmosphere. The disaster-bearing bodies of gas pipeline disasters in karst areas are the direct object that bears the hazard of disaster factors, which mainly includes social, economic and natural disaster-bearing bodies along the gas pipelines. Different disaster-bearing bodies are closely related and interact with each other. The vulnerability of disaster-bearing bodies is mainly used to measure the difficulty of their being damaged by disasters. On the one hand, it depends on the physical characteristics of the disaster-bearing bodies, such as the strength and ductility level of gas pipelines. On the other hand, it depends on the cultivated disaster-bearing ability, that is, the multiple abilities of resistance, rescue and recovery in the face of disasters. The vulnerability of disaster-bearing bodies (*c*) is closely related to the social disaster-bearing body (*k*_7_), economic disaster-bearing body (*k*_8_), natural disaster-bearing body (*k*_9_) and disaster bearing capacity (*k*_10_), as shown in Fig 3.

Specifically, the social disaster-bearing body (*k*_7_) mainly includes the human casualties (*t*_37_), the destruction of buildings and structures (*t*_38_) and the destruction of lifeline engineering (*t*_39_). The economic disaster-bearing body (*k*_8_) mainly includes the material property loss (*t*_40_), economic compensation (*t*_41_), resource waste (*t*_42_) and production and business suspension (*t*_43_). The natural disaster-bearing body (*k*_9_) mainly includes the atmospheric pollution (*t*_44_), soil hardening (*t*_45_), the damage to vegetation (*t*_46_) and the harm to animals (*t*_47_). The disaster bearing capacity (*k*_10_) mainly includes the early warning capability (*t*_48_), disaster resistance capability (*t*_49_), disaster relief capacity (*t*_50_) and recovery capacity (*t*_51_).

#### 2.1.2. Establishment of Bayesian network model

**Fig 4:**
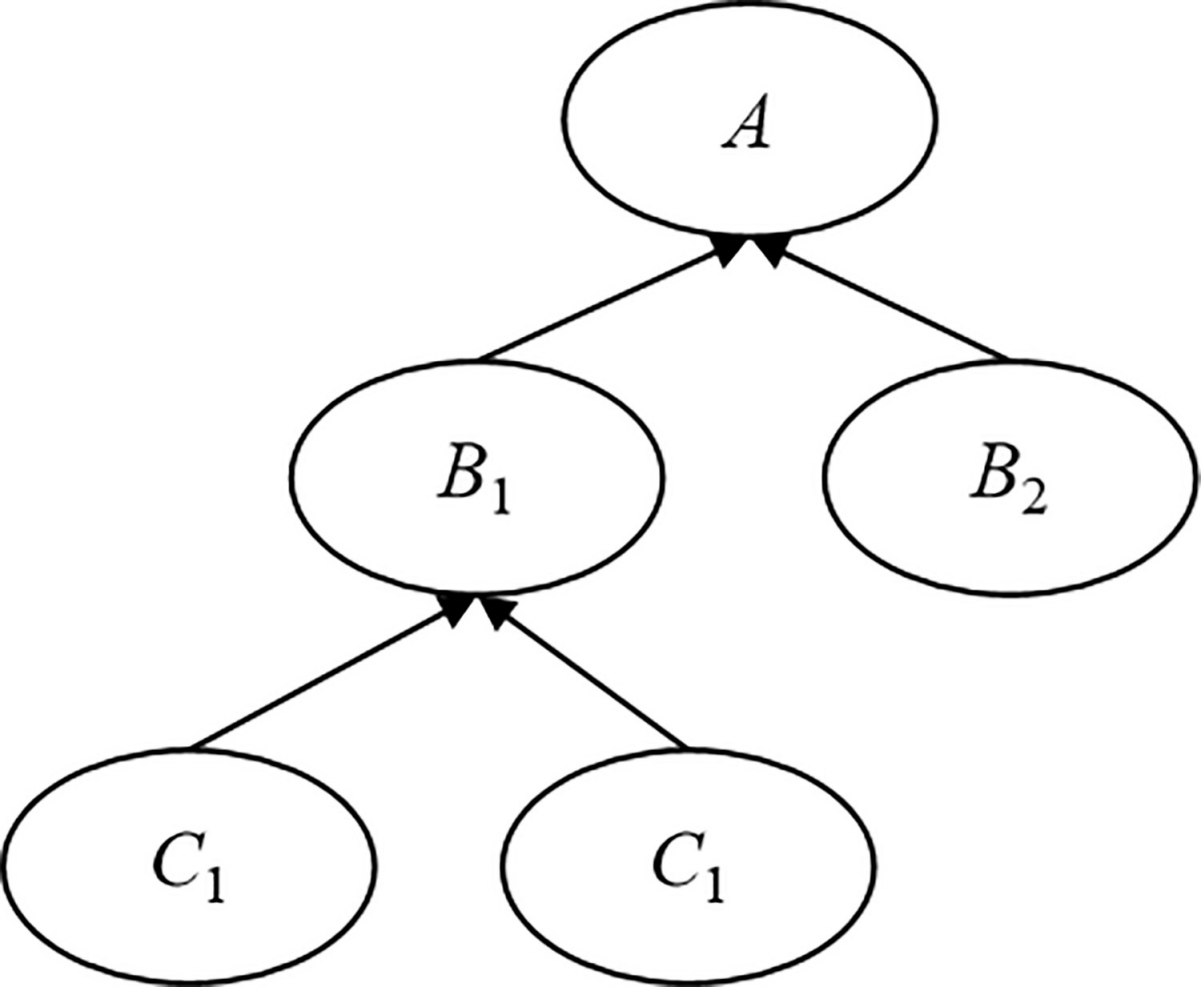
A simple Bayesian network structure.

The Bayesian network is a directed acyclic graph based on graph theory and probability theory, which is mainly used to describe the dependency relationships and probability distributions of a group of random variables. It consists of nodes, directed edges and probabilities, and a simple Bayesian network structure is shown in Fig 4.

In Fig 4, nodes *A*, *B*_1_, *B*_2_, *C*_1_ and *C*_2_ represent a group of random variables, and the arrows indicate their dependency relationships. The *C*_1_ points to *B*_1_, indicating that the *C*_1_ is the parent node of the *B*_1_, and the *B*_1_ is the child node of the *C*_1_. Meanwhile, the *C*_1_ and *C*_2_ have no parent node, so they are root nodes. The root nodes have corresponding prior probability distribution, and non-root nodes have corresponding conditional probability distribution.

The Bayesian network can update the failure probability of the system by adjusting the probability distribution of nodes. Taking Fig 4 as an example, the adjustment process of probability distribution of nodes in the Bayesian network is investigated: assuming that at a certain moment *t*, the probability that the occurrence of *C*_1_ causes the occurrence of *B*_1_ is 0.2, the probability that the occurrence of *C*_2_ causes the occurrence of *B*_1_ is 0.3. The probability of *B*_1_ occurring when *C*_1_ and *C*_2_ both occur is 0.5, and the probability of *B*_1_ occurring when neither *C*_1_ and *C*_2_ occurs is 0.1. In this regard, the conditional probability distribution corresponding to the node *B*_1_ is shown in Table 1, and 0 and 1 respectively indicate the occurrence and non-occurrence of the root nodes.

**Table 1:** The conditional probability distribution corresponding to the node *B*_1_.

Condition	Probability			
*C*_1_	0		1	
*C*_2_	0	1	0	1
*B*_1_ occurs	0.1	0.3	0.2	0.5
*B*_1_ does not occur	0.9	0.7	0.8	0.5

**Fig 5:**
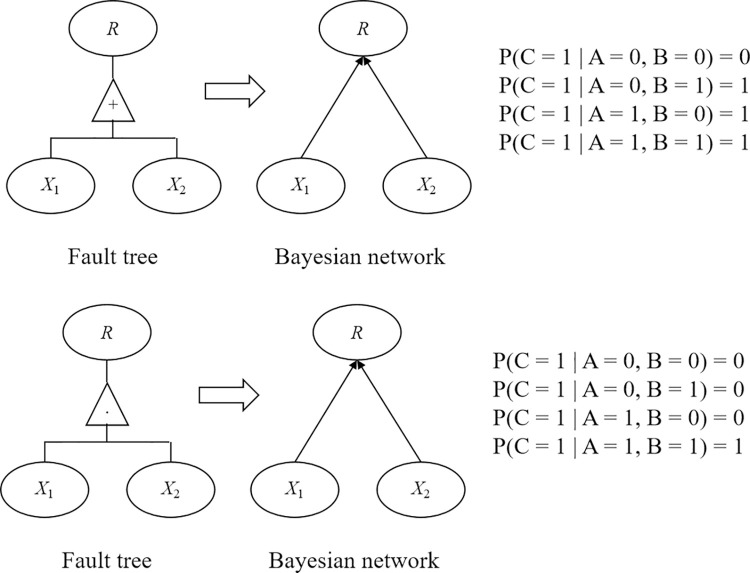
Schematic diagram of transformation between the fault tree model and the Bayesian network model. The “or” gate (A), and“and” gate (B).

In the Bayesian network model, the probability calculation of event occurrence can be equivalent to the propagation and updating of beliefs [25]. In this regard, the calculation process is simple and the results are accurate. Therefore, the Bayesian network model can be used to evaluate the hazard probability of disaster events of gas pipelines in karst areas. Meanwhile, the fault tree model and Bayesian network model have similarities in principle, and the deductive reasoning function of the fault tree model can quickly determine the dependency relationships between multiple events. In this regard, the Bayesian network model can be established based on the corresponding mapping relationship. The transformation of the “or” gate and “and” gate in the fault tree model to the Bayesian network model is shown in Fig 5.

In Fig 5, the events in the fault tree model are transformed into the nodes of the Bayesian network model, and the logical symbols are directly transformed into directed edges. Meanwhile, the dependency relationship between nodes is represented by a conditional probability table. Taking the "or" gate in the fault tree model as an example, its conditional probability table is shown in Table 2, and 0 and 1 respectively indicate the occurrence and non-occurrence of the events.

**Table 2:** The conditional probability distribution corresponding to the "or" gate.

Condition	Probability			
*X*_1_	0		1	
*X*_2_	0	1	0	1
*R* (Yes)	0.1	0.3	0.2	0.5
*R* (No)	0.9	0.7	0.8	0.5

The Bayesian network model uses probability integration to represent uncertainty, and the fundamental rule of probability integration is the probability of joint events, as shown in Formula (1).


P(A,B)=P(A|B)P(B) (1)

Where *P*(*B*) is the occurrence probability of the event *B*; *P* (*A*, *B*) is the joint probability of the event *A* and *B*; *P*(*A*|*B*) is the conditional probability of the event *A* occurring under the condition that the event *B* occurs.

By substituting the prior probability into the joint probability distribution, the occurrence probability of the highest-level event can be obtained, as shown in Formula (2).


$$P(D)=\prod_{i=1}^{n}P(X_{i}|P_{a}(X_{i}))$$ (2)

Where *P*_*a*_(*X*_*i*_) is the occurrence probability of the disaster-causing factor *X*_*i*_, which corresponds to the prior probability; *P*(*X*_*i*_|*P*_*a*_(*X*_*i*_)) is the conditional probability of the higher-level factor occurring under the condition that the disaster-causing factor *X*_*i*_ occurs; *P*(*D*) is the occurrence probability of the highest-level event, which corresponds to the hazard probability of disaster events of gas pipelines in karst areas.

#### 2.1.3. Determination of prior probability

At present, the failure database of gas pipelines in karst areas has not been established, and it is often difficult to ensure the accuracy and applicability to evaluate the occurrence probability of basic events by combining statistical analysis model. Therefore, it is necessary to determine the failure possibility of basic events based on expert experience. However, it is worth noting that even industry experts can only give some general judgments on the probability of fuzzy events that cause natural gas pipeline disasters (such as the lack of safety supervision, inadequate emergency support, etc.) [26]. In this regard, fuzzy mathematics is a good method for solving the problem of insufficient known data. In the theory of fuzzy mathematics, the index score in the evaluation system can reflect the occurrence possibility of an event. Assuming that the *V*_0_ is the maximum score, the score values of each evaluation index can be divided into five intervals, corresponding to different possibility levels [27], as shown in Table 3.

**Table 3:** The correspondence between score intervals and possibility levels.

Possibility level	Score interval
Low (L)	0 < *V* ≤ 0.2*V*_0_
Relatively low (RL)	0.2*V*_0_ < *V* ≤ 0.4*V*_0_
Medium (M)	0.4*V*_0_ < *V* ≤ 0.6*V*_0_
Relatively high (RH)	0.6*V*_0_ < *V* ≤ 0.8*V*_0_
High (H)	0.8*V*_0_ < *V* ≤ *V*_0_

**Fig 6:**
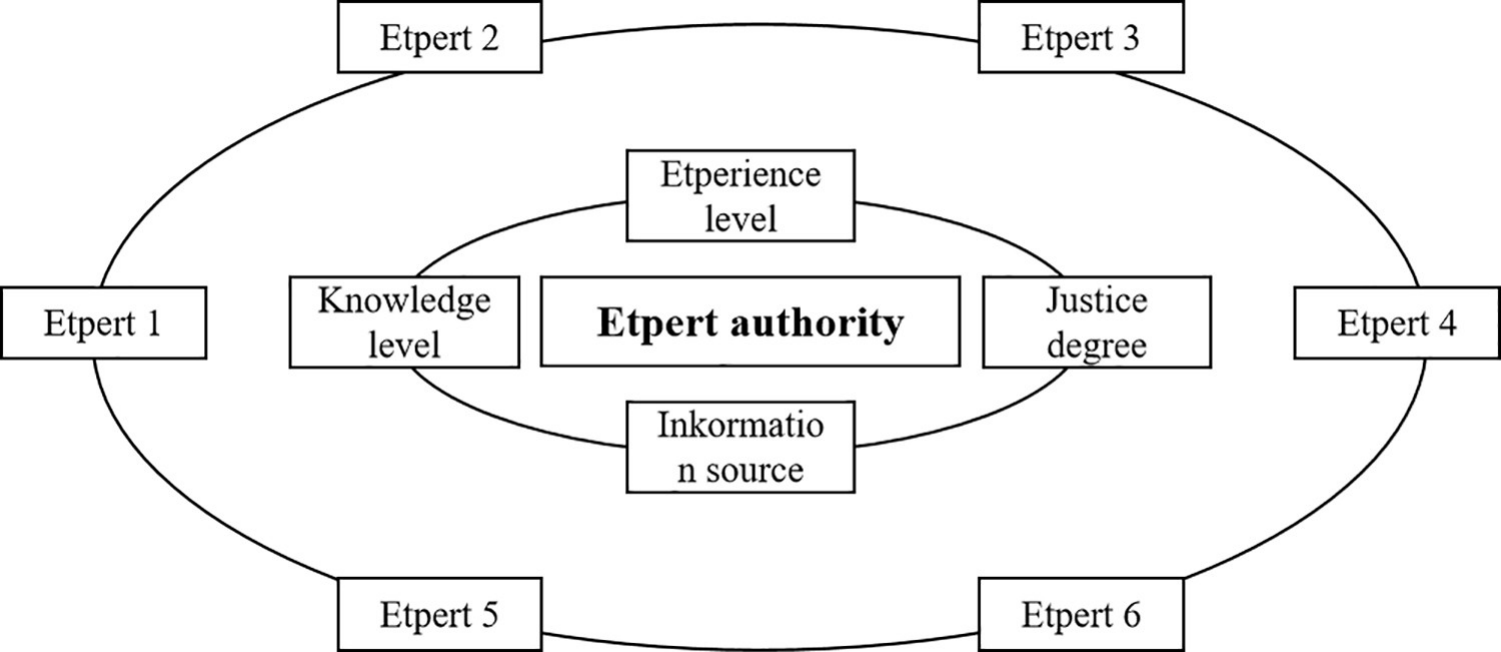
AHP model of expert authority evaluation.

Considering that the differences of experts’ knowledge level, experience level, information source and justice degree will lead to the differences of their decision-making, the analytic hierarchy process (AHP) model of expert authority evaluation is established, as shown in Fig 6.

Next, based on the judgment matrix, the influence weights of different related factors on the overall evaluation ability of experts, as well as the weights of different experts on the same evaluation ability related factors are investigated respectively. The construction principle of the evaluation matrix is that the *c*_*ij*_ in the matrix indicates the superiority of the indicator *u*_*i*_ compared with the indicator *u*_*j*_, which conforms to the setting of Formula (3).


$$\{\begin{array}{c} c_{ii}=1\\ c_{ij}•c_{ji}=1 \end{array}$$ (3)

By revising the evaluation opinions based on the weights of experts’ comprehensive ability, it is helpful to make the evaluation results more in line with the objective reality. In this study, an improved AHP model based on the 1–9 exponential scale is used to determine the expert authority weights, and its specific scale value is shown in Table 4.

**Table 4:** Exponential scale value.

Scale definition	Equally important	Slightly important	Important	Obviously important	Strongly important	Extremely important
Scale value	1	1.3161	1.7321	3	5.1966	9

Next, the weight vectors are used to represent the influence weights of different experts on the same evaluation ability related factor. Assuming that there are *n* experts in total, the *c*_*st*_ represents the superiority of the *s*-th expert over the *t*-th expert for the *i*-th evaluation ability related factor, and its normalized value is *c*’_*st*_. In this regard, the weight *W*_*is*_ of the *s*-th expert relative to the *i*-th evaluation ability related factor is shown in Formula (4).


$$W_{is}=\frac{\prod_{t=1}^{n}c_{st}^{'}}{\sum_{k=1}^{n}\left(\prod_{t=1}^{n}c_{kt}^{'}\right)}$$ (4)

**Fig 7:**
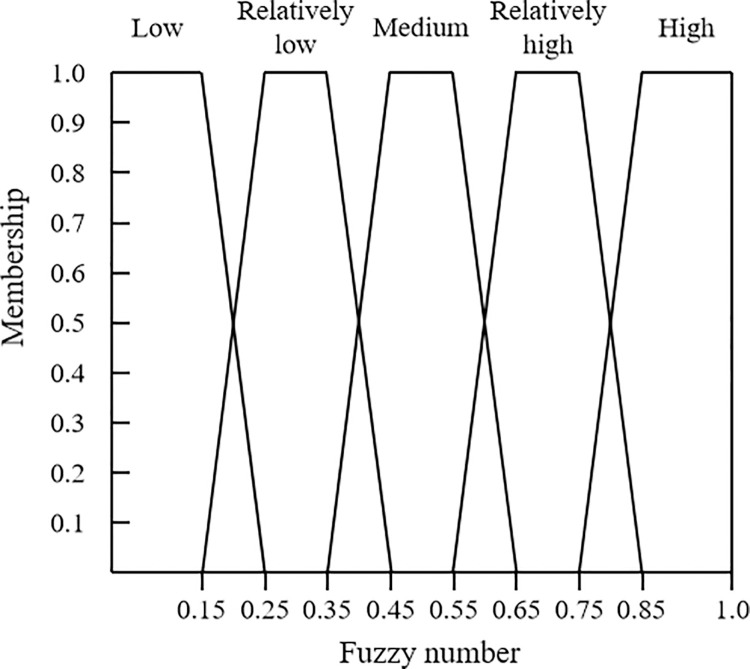
Membership functions corresponding to trapezoidal the fuzzy numbers.


The basic principle of the AHP method is to compare the importance of influencing factors. Then, based on consistency testing, the evaluation results are investigated to determine if their inconsistency exceeds the allowable range. The fuzzy set theory uses fuzzy numbers to represent the relationship between uncertain variables and membership functions. In this study, the trapezoidal fuzzy number is selected to capture the uncertainty of experts’ judgment. In this regard, the risk levels of basic events corresponding to different indexs can be further transformed into the trapezoidal fuzzy numbers [28], as shown in Fig 7. For a certain fuzzy number *M*, its corresponding membership function model is shown in Formula (5).

$$E_{M}(f)=\{\begin{array}{l} f-f_{\min}/0.1\;(f_{\min}\leq f\leq f_{\min}+0.1)\\ 1\;(f_{\min}+0.1\leq f\leq f_{\max}-0.1)\\ f_{\max}-f/0.1\;(f_{\max}-0.1<f\leq f_{\max})\\ 0\;(else) \end{array}$$ (5)

Where *f*_max_ and *f*_min_ are the upper and lower limits of the fuzzy numbers, respectively.

The *α*-cut set of fuzzy set is selected to comprehensively process the experts’ evaluation opinions, and then the corresponding fuzzy number function can be obtained. The fuzzy number function based on experts’ evaluation opinions is a fuzzy set within [0, 1]. It is necessary to convert it into a clear value, that is, fuzzy possibility score. It represents experts’ trust in the occurring of an basic event. Based on the left and right fuzzy sorting method, the fuzzy number can be converted into fuzzy possibility score [29]. The corresponding maximum fuzzy set *S*(*f*)_max_ and minimum fuzzy set *S*(*f*)_min_ are shown in Formula (6) and Formula (7) respectively.


$$S{\left(f\right)}_{\max}=\{\begin{array}{c} f(0<f<1)\\ 0(f\leq 0\;\mathrm{or}\;f\geq 1) \end{array}$$ (6)

$$S{\left(f\right)}_{\min}=\{\begin{array}{c} 1-f(0<f<1)\\ 0(f\leq 0\;\mathrm{or}\;f\geq 1) \end{array}$$ (7)

On this basis, the left and right fuzzy possibility score of the fuzzy number *M* are shown in Formula (8) and Formula (9) respectively.


$$F_{\mathrm{PS},L}(M)=\underset{x}{\sup}[E_{N}(f)\wedge S{\left(f\right)}_{\min}]$$ (8)

$$F_{\mathrm{PS},R}(M)=\underset{x}{\sup}[E_{N}(f)\wedge S{\left(f\right)}_{\max}]$$ (9)

Where sup is the supremum of the set; *E*_*N*_(*f*) is the membership function corresponding to the fuzzy number *M*; ∧ is the arithmetic operation of taking the smaller of two values in fuzzy mathematics theory.

On this basis, the fuzzy possibility score *S* of the fuzzy number *M* is shown in Formula (10).


$$S=[F_{\mathrm{PS},R}(M)+1-F_{\mathrm{PS},L}(M)]/2$$ (10)

Then, the fuzzy possibility score of each evaluation index is transformed into fuzzy failure probability. It is taken as the evaluation value of the prior probability of the corresponding disaster-causing factor, and its specific calculation is shown in Formulas (11) and (12).


$$p=\{\begin{array}{c} \frac{1}{10^{r}}\;(S\neq 0)\\ 0\;(S=0) \end{array}$$ (11)

r=(1−SS)13×2.301 (12)

### 2.2. Vulnerability analysis of gas pipeline

The vulnerability refers to the possibility that the disaster-bearing body is susceptible to the damage or destruction caused by disaster-causing factors [30]. That is, the structure failure probability under different levels of disasters. In engineering practice, the vulnerability has been given different definitions and can be generally classified into the following three categories: (1) The possibility of potential destructive events causing damage to the disaster-bearing bodies; (2) The ability of the disaster-bearing bodies to predict, respond to, and handle disasters, as well as the ability to recover after disasters; (3) The comprehensive measurement of economic and social capacity of disaster-bearing bodies to respond to disaster events and disaster risks. The vulnerability level of gas pipelines is closely related to the disaster type and intensity, pipeline structure and function, the temporal and spatial configuration relationship between the disasters and the pipelines, and the ability of human society to prevent and reduce disasters [31].

#### 2.2.1. Construction of vulnerable function based on structural reliability theory

In the field of pipeline structural reliability research, the load applied to the pipelines that causes mechanical response is called generalized load, and the ability of the pipeline structure to resist generalized load is called generalized resistance [32]. The critical state function for pipeline structural damage is defined as *D*, which is expressed as the difference between the generalized resistance and the generalized load. When the function value is greater than 0, the pipeline structure is in a safe state; When the value is equal to 0, it is in a critical state; When the value is less than 0, it will enter the invalid state. In this regard, the basic principle equation of the structure failure probability *P*_*f*_ of gas pipelines is shown in Formula (13).


$$P_{f}=P(D<0)$$ (13)

For the gas pipelines in karst areas, the vulnerability depends on the combined effects of the damage intensity of disaster load to the pipelines, and the ability of pipelines to resist disaster damage. The pipeline vulnerability probability corresponds to the structure failure probability in the structural reliability theory. Therefore, the Formula (13) is transformed to obtain the pipeline vulnerability function, as shown in Formula (14).


$$P(V>0)=P\{(I_{r}-I_{d})>0\}$$ (14)

Where *V* is the vulnerability; *I*_*r*_ is the ability of pipelines to resist disaster damage; *I*_*d*_ is the damage intensity of disaster load to the pipelines.

#### 2.2.2. Selection of evaluation index

**Fig 8:**
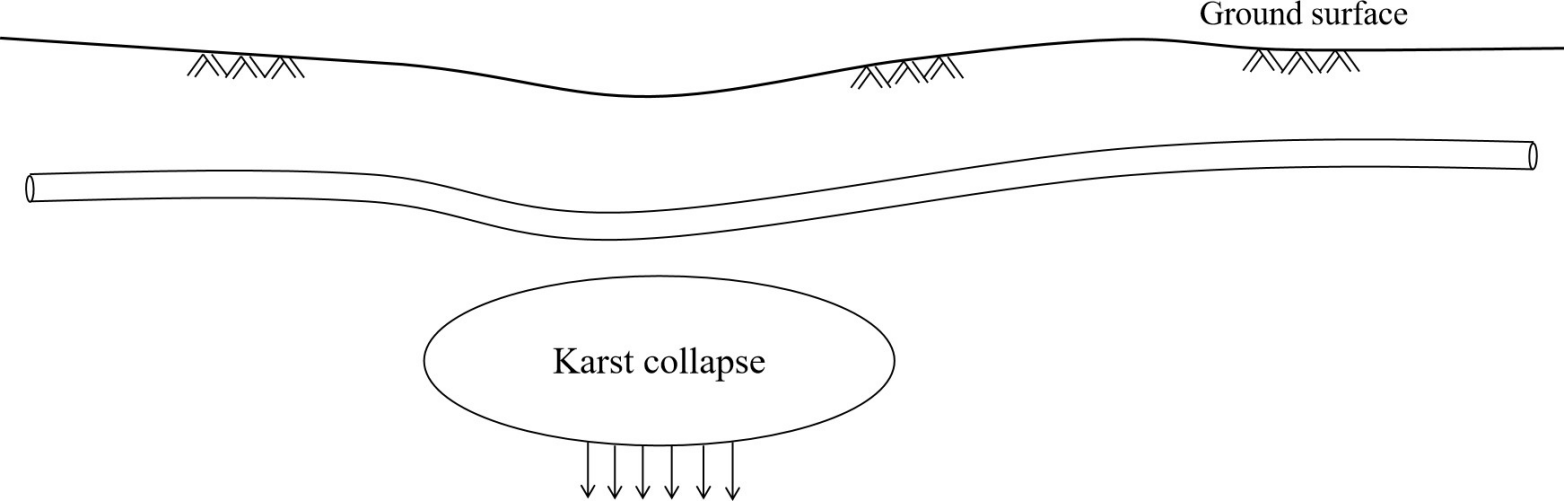
Schematic diagram of pipeline deformation under the influence of karst collapse.

Karst collapse is a kind of karst dynamic geological effect and phenomenon, and its specific development characteristics are as follows: under the influences of natural and human activities, caves and cracks develop at the top of bedrock. Their continuous upward expansion will cause the deformation of the upper rock and soil, which eventually leads to the rupture of the karst roof and the formation of surface collapse pits. In order to ensure the stability of gas pipelines and reduce the impact of external factors, gas pipelines are usually laid underground. In this regard, the deformation of buried gas pipelines will be constrained by the surrounding soil, as shown in Fig 8. Meanwhile, the mechanical parameters and deformation performance of buried pipelines are significantly different from the surrounding soil, which will lead to complex relative deformation between the pipelines and the soil in the process of karst collapse. On this basis, the variables of vulnerability function are selected from three aspects: karst collapse parameters, pipeline operation parameters and soil properties parameters, as shown in Table 5.

**Table 5:** The variables of vulnerability function.

Category	Variable	Symbol	Unit
Karst collapse parameters	The length of karst soil collapse	*K*_scl_	m
	The width of karst soil collapse	*K*_scw_	m
	The thickness of karst overburden	*K*_ot_	m
Pipeline operation parameters	The pipe wall thickness	*P*_wt_	mm
	The pipe buried depth	*P*_bd_	m
	The pipe service life	*L*_ps_	year
soil property parameters	The soil elastic modulus	*S*_em_	Mpa
	The friction coefficient between pipeline and soil	*K*_ot_	-

Based on the engineering practice and previous research [33], the following influence laws are summarized: firstly, for the karst collapse parameters, the greater the length and the width of karst soil collapse, the greater the pipeline displacement caused by soil dislocation, and the greater the soil load on the pipelines. Under the action of external force, buried pipelines will produce certain plastic deformation, especially in internal and external defects and weak structures. The stability of buried pipelines is greatly affected, and the service life of pipelines will be significantly reduced if they continue to be used for a long time. The greater the thickness of karst overburden, the more stable the upper soil structure. In this regard, the influence of underground karst collapse on the buried pipelines will weaken in the process of upward conduction, so the mechanical response of pipelines under the impact of soil load will be reduced. Secondly, for the pipeline operation parameters, the greater the wall thickness, the smaller the diameter-thickness ratio, and the greater the pipeline stiffness. The increase of pipe buried depth is beneficial to strengthen the soil arching effect and reduce the karst collapse range, thus reducing its damage capacity. However, karst soil has a strong compressive effect on deeply buried gas pipelines, leading to a sharp increase in compressive stress. Meanwhile, deep buried gas pipelines are more easily affected by the dislocation of underground soil. With the increase of service time year by year, the aging phenomenon will lead to a significant decline in pipeline strength, so service time is a key parameter affecting the vulnerability of pipelines. Thirdly, for the soil property parameters, the increase of elastic modulus of soil will reduce the deformation ability of soil and increase the soil reaction force. Under the constraint of large soil reaction forces, the compression effect on the pipelines will increase, making it difficult to utilize the adaptability of gas pipeline ductility to soil dislocation. A small friction coefficient is beneficial for reducing the soil constraints on buried pipelines under the influence of karst collapse and minimizing mutual friction. In this regard, it can educe the stress concentration of dangerous sections of buried pipelines in karst collapse areas.

For the selected eight variables, it is assumed that the critical parameters that lead to the failure of gas pipelines in karst areas are *K*_scl, *c*_, *K*_scw, *c*_, *K*_ot, *c*_, *P*_wt, *c*_, *P*_bd, *c*_, *S*_em, *c*_, *F*_*c*, *c*_ and *L*_ps, *c*_respectively in a specific research scenario. When the value of a variable is equal to its corresponding critical parameter, the gas pipeline will be in a critical failure state. On this basis, the vulnerability parameters of different variables are defined as *a*_1_, *a*_2_, *a*_3_, *a*_4_, *a*_5_, *a*_6_, *a*_7_ and *a*_8_ respectively. Taking the length of karst soil collapse *K*_scl, *c*_ as an example, its vulnerability parameter is shown in Formula (15). Then, the vulnerability probability function can be further transformed into Formula (16).


$$a_{1}=\frac{K_{\mathrm{scl}}}{K_{\mathrm{scl},c}}$$ (15)

$$V=f(I_{r},I_{d})=\frac{a_{1}a_{2}a_{7}a_{8}}{a_{3}}-\frac{a_{4}a_{5}}{a_{6}}$$ (16)

#### 2.2.3. Calculation of vulnerability probability

In this study, the vulnerability probability is calculated based on reliability evaluation method. The commonly used reliability calculation methods include second moment method, Monte Carlo method, etc. On the premise that the distribution of random variables is not clear, the first order second moment method uses the mean and standard deviation of random variables to perform linear Taylor series expansion on the functional function. The first order second moment method mainly includes the central point method and the design checkpoint method. The central point method is simple to calculate, but it is difficult to obtain the consistent reliability index for the functional functions with the same meaning but different mathematical expressions. In this regard, the design checkpoint point method can compensate for the deficiency. Meanwhile, the function expansion of the design checkpoint method at the average value is reasonable, so it can be chosen to calculate the vulnerability probability of gas pipelines in karst areas.

Firstly, the reliability index *β* is introduced. Then, it is assumed that the vulnerability function *V* obeys a normal distribution, with an average value of *μ*_*v*_ and a standard deviation of *σ*_*v*_. In this regard, the vulnerability probability function is shown in Formula (17).


$$P(V)=\int_{-\infty }^{0}f_{V}(v)dv=\int_{-\infty }^{0}\frac{1}{\sqrt{2\pi }\sigma_{v}}\exp[-\frac{{\left(v-\mu_{v}\right)}^{2}}{2{\sigma_{v}}^{2}}]dv$$ (17)

If *v* = *μ*_*v*_ + *σ*_*v*_*u*, then *dv* = *σ*_*v*_*du*. When *v* = 0, then *u* = -*μ*_*v*_/*σ*_*v*_. When *v* → -∞, then *u* → -∞. In this regard, the vulnerability probability function can be further transformed into Formula (18).


$$P(V)=\int_{-\infty }^{\frac{\mu_{v}}{\sigma_{v}}}\frac{1}{\sqrt{2\pi }}\exp[-\frac{u^{2}}{2}]du=\Phi \left(-\frac{\mu_{v}}{\sigma_{v}}\right)=\Phi (-\beta )=1-\Phi (\beta )$$ (18)

Where *Φ*(*β*) is the cumulative function of standard normal distribution; *β* is the pipeline reliability index, and *β = μ*_*v*_/*σ*_*v*_.

For the general form of vulnerability function:

$$V=g_{v}(v_{1},v_{2},\ldots ,v_{n})$$ (19)

Where *v*_1_, *v*_2_, …, *v*_n_ are *n* independent normal random variables, with an average of *μ*_*j*_ (*j* = 1, 2, …, *n*) and a standard deviation of *σ*_*j*_ (*j* = 1, 2, …, *n*).

Assuming that a design checkpoint is represented as v* = (*v*_1_*, *v*_2_*, …, *v*_n_*), and *g*(v*) is equal to 0. Then, the functional function is expanded according to the Taylor series, and its first order expansion is shown in Formula (20).


$$M\approx M_{L}=g_{v}({v_{1}}^{*},{v_{2}}^{*},\ldots ,{v_{n}}^{*})+\sum_{c=1}^{n}\frac{\partial g_{v}}{\partial v_{c}}(v_{c}-v_{c}^{*})$$ (20)

The mean and standard deviation of *M*_*L*_ are shown in Formula (21) and Formula (22).


$$\mu_{M_{L}}=g_{v}({v_{1}}^{*},{v_{2}}^{*},\ldots ,{v_{n}}^{*})+\sum_{c=1}^{n}\frac{\partial g_{v}}{\partial v_{c}}(\mu_{v_{c}}-v_{c}^{*})$$ (21)

$$\sigma_{M_{L}}=\sqrt{{\sum_{c=1}^{n}\left[\frac{\partial g_{v}}{\partial v_{c}}\times \sigma_{v_{c}}\right]}^{2}}$$ (22)

On this basis, the expression of reliability index *β* is shown in Formula (23).


$$\beta =\frac{\mu_{M_{L}}}{\sigma_{M_{L}}}=\frac{g_{v}({v_{1}}^{*},{v_{2}}^{*},\ldots ,{v_{n}}^{*})+\sum_{c=1}^{n}\frac{\partial g_{v}}{\partial v_{c}}(\mu_{v_{c}}-v_{c}^{*})}{\sqrt{{\sum_{c=1}^{n}\left[\frac{\partial g_{v}}{\partial v_{c}}\times \sigma_{v_{c}}\right]}^{2}}}$$ (23)

The disaster-causing possibility of gas pipelines in karst areas is comprehensively investigated from two dimensions: the hazard of disaster events and the vulnerability of gas pipelines. If the disaster-causing probability is *P*, then *P* = *P*(*D*)*P*(*V*). According to the standard "API 581–2008 Risk-based Inspection Technology" issued by the American Petroleum Institute (API) and "DNV-RP-F116 Integrity Management of Submarine Pipeline Systems" issued by the Det Norske Veritas (DNV), the disaster-causing probability of gas pipelines in karst areas is divided into five grades, as shown in Table 6.

**Table 6:** Classification criteria for disaster-causing probability.

Level	Possibility description	Probability range
Ⅰ	It can’t happen	<0.0001
Ⅱ	It rarely happens	[0.0001, 0.001)
Ⅲ	It happens occasionally	[0.001, 0.01)
Ⅳ	It may happen	[0.01, 0.1)
Ⅴ	It happens frequently	>0.1

## 3. Case analysis

Zhijin County, Guizhou Province, China is located at the intersection of the Nayong-Kaiyang east-west structural belt and the Zhijin northeast structural belt. Under the joint sculpture of internal and external forces, various karst landscapes have been formed in the region. Under the influence of multiple factors such as disorderly mining, underground cave development, sustained heavy rainfall, and seismic activity, karst collapse occurs frequently. A buried gas pipeline project in Zhijin county is selected as the study case.

### 3.1 Calculation of hazard probability

In this study, six experts from different fields such as gas pipeline design, construction, management, safety risk assessment, disaster rescue, disaster prevention and control are hired to form an expert evaluation team. The descriptive statistics of the experts are shown in Table 7.

**Table 7:** Descriptive statistics of the experts.

Dimension	Attribute	Percentage
Gender	Male	16.67%
	Female	83.33%
Age	31 ~ 40 years old	16.67%
	41 ~ 50 years old	50.00%
	Over 51 years old	33.33%
Education	Undergraduate	16.67%
	Master	50.00%
	Doctor	33.33%
Personnel nature	Enterprise staff	33.33%
	Government staff	33.33%
	Research staff	33.33%

Then, the optimization matrixes of knowledge level, experience level, information source and justice degree of different experts are constructed (Data in S1 File). Through the hierarchical analysis of expert authority evaluation, the evaluation ability weights of each expert are obtained as *w* = (*w*_*e*1_, *w*_*e*2_, *w*_*e*3_, *w*_*e*4_, *w*_*e*5_, *w*_*e*6_) = (0.1720, 0.1422, 0.1715, 0.1928, 0.1441, 0.1773). Then, all the experts are hired to evaluate the hazard level for various basic events based on the knowledge and experience in their respective fields of work (Data in S2 File). On this basis, the fuzzy evaluation language of the experts is quantified based on the membership function, so as to investigate the fuzzy probability of various basic events. In addition, considering that the events related to karst collapse are in a critical position in the hazard evolution of gas pipeline disasters, the risk level of basic events *t*_15_~*t*_19_ is classified combined with the actual exploration data in karst areas. Meanwhile, micro concretization is carried out for each basic event, and the overall evaluation results are obtained through weighted average processing. Through this approach, it is helpful to more accurately evaluate the influence of key disaster events on hazard probability combined with engineering field data. The specific evaluation criteria of events related to karst collapse are shown in Table 8.

**Table 8:** The specific evaluation criteria of events related to karst collapse.

Disaster event		Low (L)	Relatively low (RL)	Medium (M)	Relatively high (RH)	High (H)
Karst geological development (*t*_15_)	Bedrock lithology	-	Sandstone and mudstone	Dolomite	Calcite dolomite	Limestone
	Karst development degree	Underdevelopment	Weak development	Moderate development	Relatively strong development	Strong development
Groundwater activity (*t*_16_)	Vertical distance between groundwater and bedrock surface	> 20m	15 ~ 20m	10 ~ 15m	5 ~ 10m	< 5m
	Variation range of groundwater	< 0.5m	0.5 ~ 1m	1 ~ 1.5m	1.5 ~ 2m	> 2m
Overburden characteristics (*t*_17_)	Overburden thickness	> 10m	8 ~ 10m	5 ~ 8m	3 ~ 5m	< 3m
	Overburden structure	Unstructured	Multivariate structure	Dual structure	Unitary structure	Hybrid structure
Structural conditions (*t*_18_)	Fault property	Compressive fault	Transpressional fault	Wrench fault	Transtensional fault	Extensional fault
	Distance from fault	> 500m	300 ~ 500m	200 ~ 300m	100 ~ 200m	< 100m
Topographic features (*t*_19_)		Mountainous region	Slope land	Flat terrain	Low lying land	Trench terrain

Taking "*t*_1_ Low level of education" as an example, the evaluation results of the expert evaluation team on the hazard level of this basic event are L, L, RL, RL, L and RL respectively. Based on the Formula (5), the membership functions of L and RL are constructed as shown in Formula (24) and Formula (25) respectively. On this basis, the corresponding *α*-cut sets are *E*_*W*_^*α*^ = [0.15+0.1α, 0.45–0.1α] and *E*_*S*_^*α*^ = [0.55+0.1α, 0.85–0.1α] respectively.


$$f_{L}(x)=\{\begin{array}{c} 1\\ \frac{0.25-x}{0.1}\\ 0 \end{array}\;\begin{array}{c} \left(0\leq x\leq 0.15\right)\\ \left(0.15\leq x\leq 0.25\right)\\ (else) \end{array}$$ (24)

$$f_{\mathrm{RL}}(x)=\{\begin{array}{c} \frac{x-0.15}{0.1}\\ 1\\ \frac{0.45-x}{0.1}\\ 0 \end{array}\;\begin{array}{c} \left(0.15\leq x\leq 0.25\right)\\ \left(0.25\leq x\leq 0.35\right)\\ \left(0.35\leq x\leq 0.45\right)\\ \left(else\right) \end{array}$$ (25)

The fuzzy number *M* considering the expert evaluation authority is further obtained as shown in Formula (26).


$$\begin{array}{l} M=\max[w_{e1}{f_{L}}^{\alpha }\wedge w_{e2}{f_{L}}^{\alpha }\wedge w_{e3}{f_{RL}}^{\alpha }\wedge w_{e4}{f_{RL}}^{\alpha }\wedge w_{e5}{f_{L}}^{\alpha }\wedge w_{e6}{f_{RL}}^{\alpha }]\\ \;=[0.1720\times 0+0.1422\times 0+0.1715\times (0.15+0.1\alpha )+0.1928\times (0.15+0.1\alpha )+0.1441\times 0+0.1773\times (0.15+0.1\alpha ),\\ \;0.1720\times (0.25-0.1\alpha )+0.1422\times (0.25-0.1\alpha )+0.1715\times (0.45-0.1\alpha )+0.1928\times (0.45-0.1\alpha )+\\ \;0.1441\times (0.25-0.1\alpha )+0.1773\times (0.45-0.1\alpha )]=[0.081252+0.054168\alpha ,0.358336-0.1\alpha ] \end{array}$$ (26)

Combined with fuzzy set extension theory, the comprehensive evaluation function of the fuzzy number *M* considering the weights of different experts is shown in Formula (27).


$$E_{M}(f)=\{\begin{array}{c} \frac{x-0.081252}{0.054168},\;(0.081252<x\leq 0.135420)\\ 1,\;(0.135420<x\leq 0.258336)\\ \frac{0.358336-x}{0.1},\;(0.258336<x\leq 0.358336)\\ 0,\;(else) \end{array}$$ (27)

**Fig 9:**
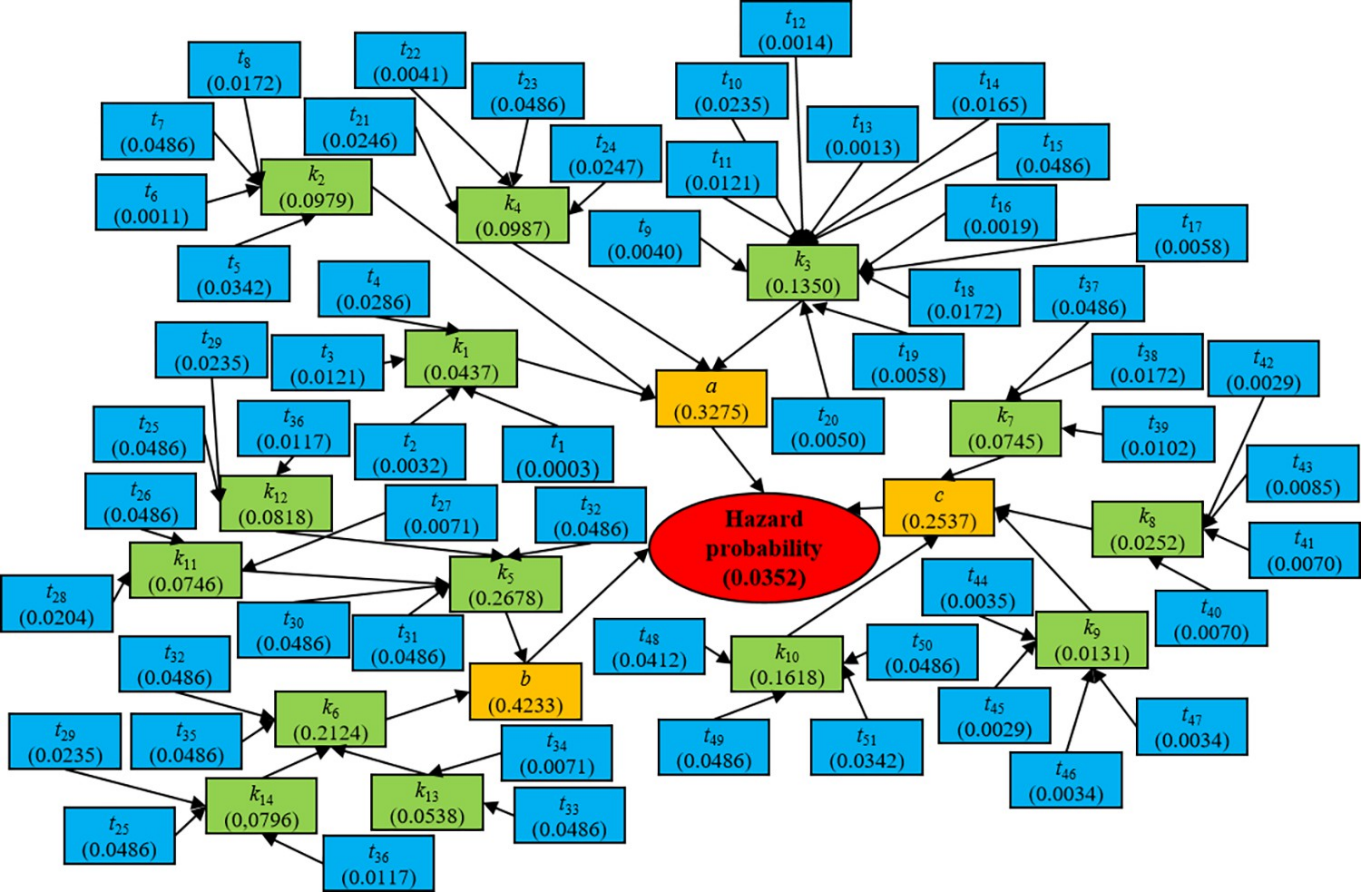
Calculation results of hazard probability based on the Bayesian network model.

Based on the Formula (6) to Formula (10), the left fuzzy possibility value *F*_SP,*L*_(*M*) is calculated to be 0.8715, and the right fuzzy possibility value *F*_SP,*R*_(*M*) is calculated to be 0.3258. Combining the left and right fuzzy possibility values, the fuzzy possibility score is calculated to be 0.2271. Based on the Formula (11) and Formula (12), the prior probability value *p*_*j*_(1) of the basic event "*t*_1_ Low level of education" is calculated to be 0.0003. Similarly, the prior probabilities of other disaster events are calculated. Then, according to the Bayesian network calculation rule, the probability distribution of nodes at all levels is calculated step by step. The specific results are summarized in Fig 9. Finally, the hazard probability *P*(*D*) of gas pipeline disaster events in the study area is calculated to be 0.0352.

### 3.2 Calculation of vulnerability probability

**Fig 10:**
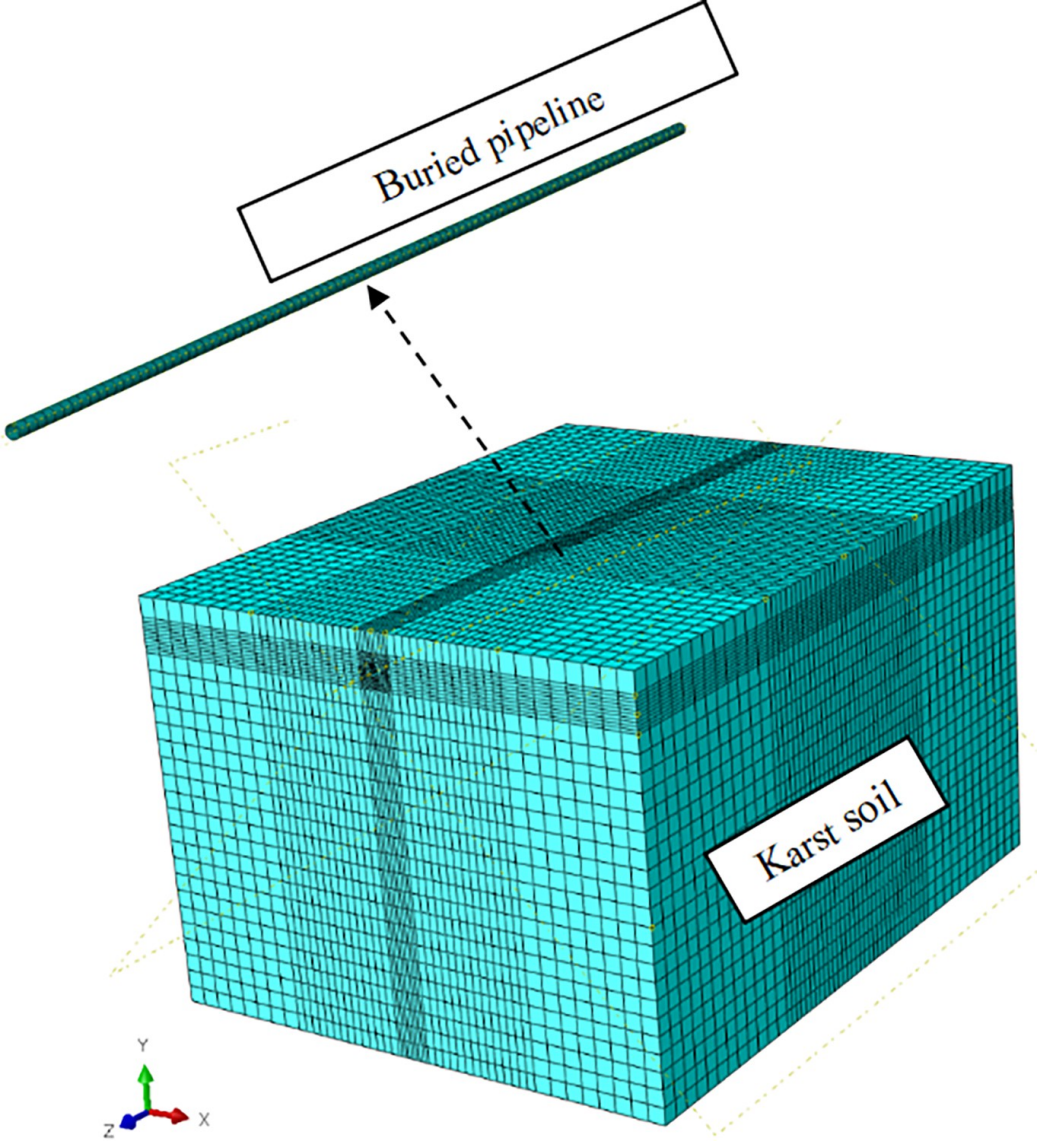
Finite element model of buried pipeline in karst areas.

In order to determine the critical value of each vulnerability index, the finite element model of buried pipelines in karst areas is established based on the ABAQUS software, as shown in Fig 10. Considering that gas pipelines mainly adopt thin-walled structures, the 4-node shell elements are used for pipeline modeling to simulate their actual characteristics. In order to ensure the simulation accuracy and control the calculation cost, the pipeline finite element model is divided into 100 units in the longitudinal direction and 30 units in the circumferential direction, totaling 3000 shell units. The basic parameters of PE80 pipeline are shown in Table 9.

**Table 9:** The basic parameters of PE80 pipeline.

Pipe Material	Pipe density (kg/m^3^)	pipe diameter (mm)	Pipe wall thickness (mm)	Transient relaxation modulus (MPa)	Poisson’s ratio	Design stress (MPa)
PE80	950	200	18.20	578.71	0.40	4

When the soil is in the elastic-plastic stage, it will show the nonlinear characteristics of materials under the multiple influences of external environment, gravity and pipeline reaction force. Therefore, the Mohr-Coulomb model is chosen to simulate soil properties. In addition, the 8-node 3D solid elements are used for soil modeling. They have the characteristics of elastoplasticity and large displacement, and conforms to the characteristics of karst collapse. According to the study on design parameters by the American Society of Mechanical Engineers [34], the effective calculation length, width, and height of the soil model are set to 20m, 16m, and 12m, respectively. Meanwhile, the structured grid division technology is used to mesh the soil elements within the vertical and horizontal 1m of the pipeline, and finally the soil model is divided into 127,008 solid elements. The property parameters of the soil model are based on the field exploration data from karst areas in Guizhou Province, China. Considering the complex and variable geological structure of karst areas, the soil layers are simplified into three categories: plain fill, clay, and bedrock. The specific parameters are shown in Table 10.

**Table 10:** The property parameters of soil model.

Soil type	Elastic modulus (MPa)	Poisson’s ratio	Soil density (kg/m^3^)	Expansion angle (°)	Cohesion force (kPa)	Internal friction angle (°)
plain fill	15	0.40	1837	0	15	15
Clay	25	0.39	1939	0	20	20
bedrock	600	0.25	2600	12	500	35

**Fig 11:**
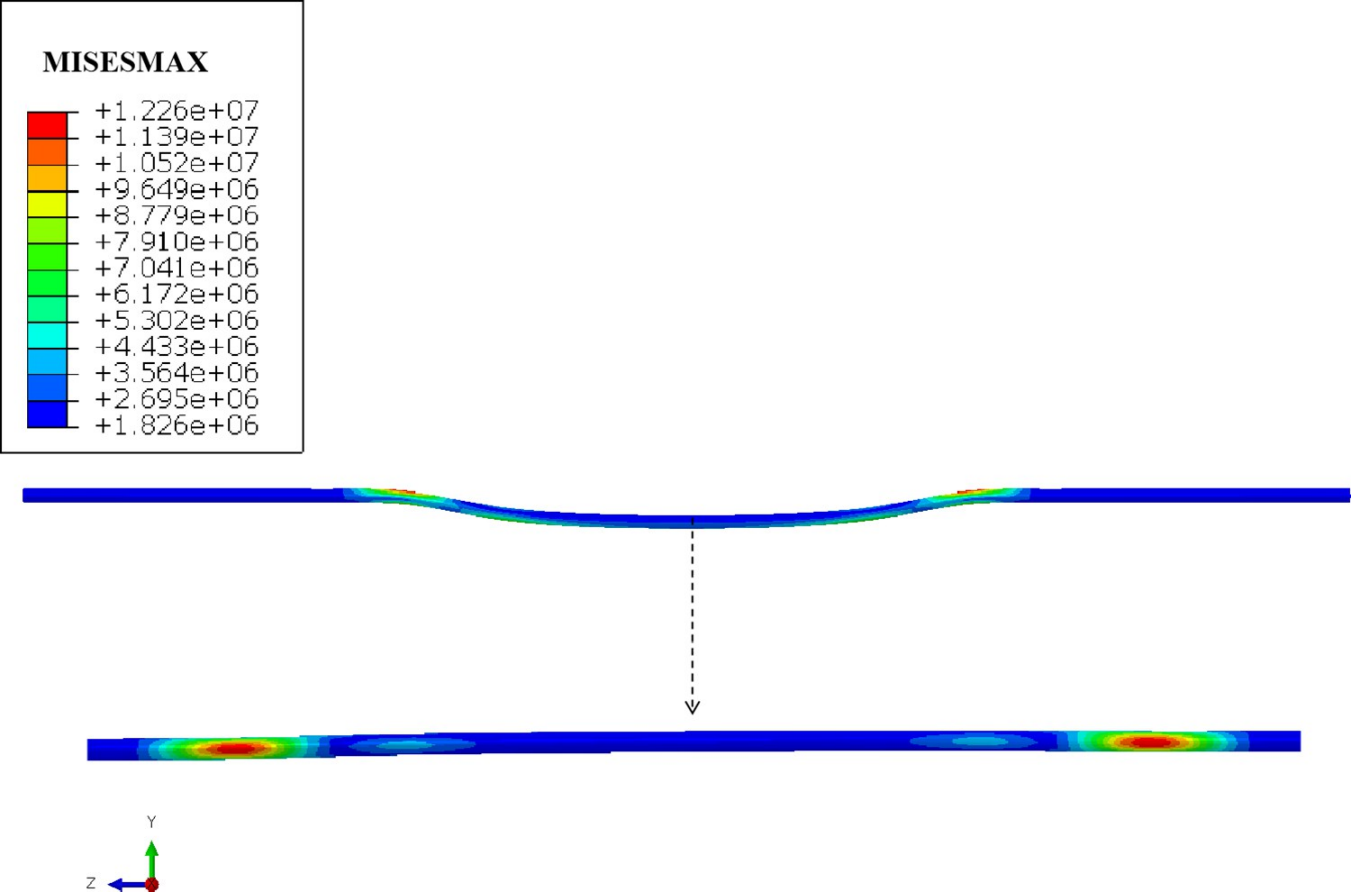
The mechanical characteristics of buried gas pipeline under the influence of karst collapse.

In the process of karst collapse, the collapse range of the upper soil layer gradually expands with the development of roof cracks. That is, the supporting force on the karst overburden near the crack gradually decreases in the vertical direction, rather than the whole soil directly moving downwards. The element birth and death technique provided by the ABAQUS finite element analysis software can achieve the "killing" and reactivation of soil elements, thereby accurately simulating the soil loss during the gradual development of karst collapse. Therefore, the karst soil in the lower part of the pipeline is divided into five unit sets from inside to outside along the collapse direction, and then the "Invalid in this step" is set in the software interaction attribute module. On this basis, the gradual development characteristics of karst collapse from the central soil cave to both sides are simulated based on the element birth and death technique. The mechanical characteristics of buried gas pipeline under the influence of karst collapse are shown in Fig 11.

Based on the finite element simulation results, the value ranges of different vulnerability parameters are further analyzed:

(1) Karst collapse parameters

When the karst collapse load directly affects the PE80 pipeline with a diameter of 200mm, a wall thickness of 18.2mm and a buried depth of 1m, the critical length of karst soil collapse that caused the pipeline failure is 5.6m, the critical width of karst soil collapse is 6.1m, and the critical thickness of karst overburden is 2.6m. At this time, for Formula (16), *a*_1_ = *K*_scl_/5.6, *a*_2_ = *K*_scw_/6.1 and *a*_3_ = *K*_ot_/2.6. Combined with the karst exploration data along the gas pipelines in the study area, the length of karst soil cave ranges from 2 to 10m, the width of karst soil cave ranges from 0.3 to 6.5m, and the thickness of karst overburden ranges from 1.8 to 19.3m. In this regard, the value ranges of *a*_1_, *a*_2_ and *a*_3_ can be obtained. It is assumed that the *a*_1_, *a*_2_ and *a*_3_ are randomly distributed in their respective value ranges, and 10000 random numbers are generated in their value ranges respectively based on the Matlab programming (Data in S3 File). The average values and standard deviations of each group of random numbers are calculated and summarized in Table 11.

**Table 11:** Related values of vulnerability parameters.

Variables	Vulnerable parameter	Critical value	Value range	Average value	Standard deviation
*K*_scl_ (m)	*a*_1_	5.60	[2/5.60, 10/5.60]	1.07	0.41
*K*_scw_ (m)	*a*_2_	6.10	[0.30/6.10, 6.50/6.10]	0.55	0.29
*K*_ot_ (m)	*a*_3_	2.60	[1.80/2.60, 19.30/2.60]	0.79	0.38
*P*_wt_ (mm)	*a*_4_	16.89	[16.20/16.89, 20.20/16.89]	0.94	0.06
*P*_bd_ (m)	*a*_5_	0.97	[0.60/0.97, 1.50/0.97]	1.13	0.28
*S*_em_ (MPa)	*a*_6_	22.63	[15/22.63, 25/22.63]	0.88	0.13
*F*_*c*_	*a*_7_	0.45	[0.30/0.45, 0.50/0.45]	0.89	0.13
*L*_ps_ (year)	*a*_8_	50	[4/50, 1]	0.54	0.27

(2) Pipeline operation parameters

When the karst collapse load directly affects the PE80 pipeline with a diameter of 200mm, and a buried depth of 1m, the critical pipe wall thickness is 16.89mm. At this time, for Formula (16), *a*_4_ = *P*_wt_/16.88. According to the national standard of the People’s Republic of China, "GB 15558.1 Buried Polyethylene (PE) Pipeline Systems for Gas Use Part 1: Pipes", the minimum wall thickness of SDR11 series pipeline materials is 18.2mm, and the deviation between the wall thickness at any point and the minimum wall thickness is within 2mm. Therefore, the pipe wall thickness ranges from 16.2 to 20.2mm. In this regard, the value range of the *a*_4_ can be obtained. It is assumed that the *a*_4_ is randomly distributed in its respective value range, and 10000 random numbers are generated in its value range based on the Matlab programming (Data in S3 File). The average value and standard deviation of the group of random numbers are calculated and summarized in Table 11.

When the karst collapse load directly affects the PE80 pipeline with a diameter of 200mm and a wall thickness of 18.2mm, the critical pipe buried depth that caused the pipeline failure is 0.97m. At this time, for Formula (16), *a*_5_ = *P*_bd_/0.97. Due to the complex and changeable geological environment along the gas pipeline in the study area, the buried depth of some pipeline sections exceeds or is less than the standard depth by about 1m during the process of actual laying. On the whole, the buried depth is in the range of 0.6 ~ 1.5m. In this regard, the value range of the *a*_5_ can be obtained. It is assumed that the *a*_5_ is randomly distributed in its respective value range, and 10000 random numbers are generated in its value range based on the Matlab programming (Data in S3 File). The average value and standard deviation of the group of random numbers are calculated and summarized in Table 11.

According to the national standard of the People’s Republic of China, "GB 50494–2009 Technical Specification for Urban Gas", the design service life of gas pipelines in China should be greater than 30 years, and the service life of PE pipelines can generally reach 40 ~ 50 years. Therefore, the service life of PE gas pipelines is taken as 50 years in this study. At this time, for Formula (16), *a*_8_ = *L*_ps_/50. The PE gas pipeline in the study area has been in service for 4 years. In this regard, the value range of the *a*_8_ can be obtained. It is assumed that the *a*_8_ is randomly distributed in its respective value range, and 10000 random numbers are generated in its value range based on the Matlab programming (Data in S3 File). The average value and standard deviation of the group of random numbers are calculated and summarized in Table 11.

(3) Soil property parameters

When the karst collapse load directly affects the PE80 pipeline with a diameter of 200mm, a wall thickness of 18.2mm and a buried depth of 1m, the critical soil elastic modulus is 22.63MPa. At this time, for Formula (16), *a*_6_ = *S*_em_/22.63. Due to the complex geological structure along the gas pipeline in the study area, the soil layer where the buried pipeline is located is mainly composed of plain fill and clay, and its elastic modulus is mainly in the range of 15 ~ 25MPa. In this regard, the value range of the *a*_6_ can be obtained. It is assumed that the *a*_6_ is randomly distributed in its respective value range, and 10000 random numbers are generated in its value range based on the Matlab programming (Data in S3 File). The average value and standard deviation of the group of random numbers are calculated and summarized in Table 11.

When the karst collapse load directly affects the PE80 pipeline with a diameter of 200mm, a wall thickness of 18.2mm and a buried depth of 1m, the critical friction coefficient between pipeline and soil is 0.45. At this time, for Formula (16), *a*_7_ = *F*_*c*_/0.45. Due to the complex and variable soil properties and moisture content along the gas pipeline in the study area, the friction coefficient is mainly in the range of 0.3 ~ 0.5. In this regard, the value range of the *a*_7_ can be obtained. It is assumed that the *a*_7_ is randomly distributed in its respective value range, and 10000 random numbers are generated in its value range based on the Matlab programming (Data in S3 File). The average value and standard deviation of the group of random numbers are calculated and summarized in Table 11.

**Fig 12:**
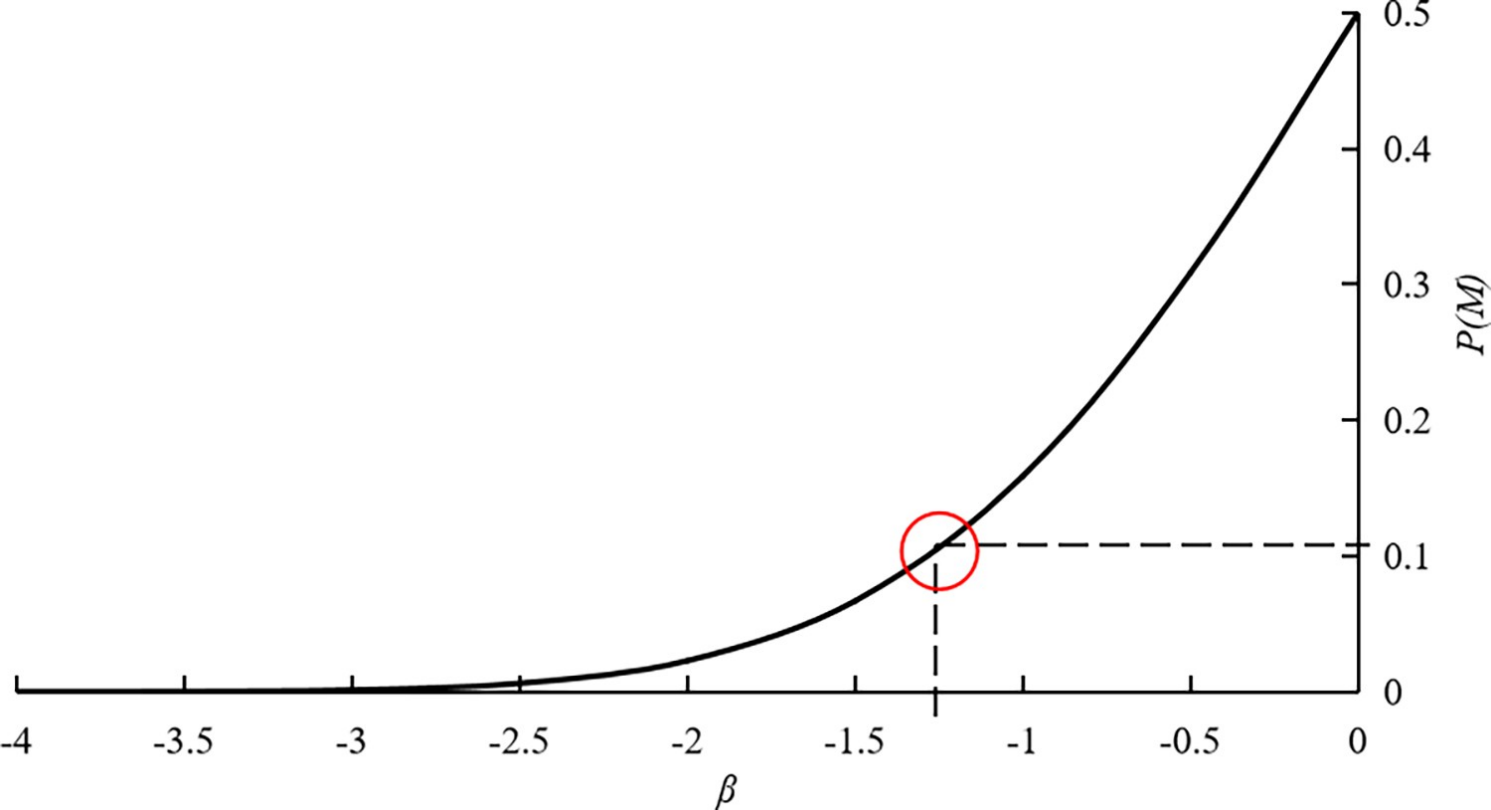
Relationship between the vulnerability probability *P*(*V*) and the reliability index *β*.

The initial design checkpoint is set based on the average value of each vulnerability parameters, that is, v*(0) = (1.07, 0.55, 0.79, 0.94, 1.13, 0.54, 0.88, 0.89). Based on the Formula (17) to Formula (23), the corresponding calculation program is compiled based on the MATLAB programming. The iteration number of model calculation is 13, and the reliability index *β* is finally calculated to be -1.2639. By consulting the standard normal distribution table, the vulnerability probability *P*(*V*) of gas pipelines in karst areas based on the design checkpoint method is 0.1038, as shown in Fig 12 [35].

In this study, the disaster-causing possibility of gas pipelines in karst areas is comprehensively measured from two dimensions: the hazard of disaster events and the vulnerability of gas pipelines. Therefore, the disaster-causing probability is as follows: *P* = *P*(*D*)*P*(*V*) = 0.0352 × 0.1038 = 0.0037. According to the classification criteria, the disaster-causing probability is at level III (It happens occasionally). This level is unacceptable for the daily operation of gas pipelines, so it is necessary to take corresponding disaster prevention and control measures to reduce the risk of disaster events and the vulnerability of gas pipelines in karst areas.

## 4. Conclusion

(1) Based on the disaster system theory, the hazard evaluation index system of gas pipeline disaster events in karst areas is established from three aspects: the activity of disaster-prone environment, the risk of disaster factors and the vulnerability of disaster-bearing bodies. Meanwhile, based on the Bayesian network model, the calculation of the occurrence probability of basic events is equivalent to the propagation and renewal of beliefs, which can make up for the limitations of effective calculation bits and complicated calculation process of fault tree model. Therefore, it is helpful to measure the hazard probability of disaster events more simply and accurately from a systematic perspective.

(2) Considering the pipeline vulnerability level and its relationship with the disaster type and intensity, pipeline structure and function, disaster and pipeline space-time configuration, a mathematical model system is established based on the polyethylene pipeline characteristics. Combined with the design checkpoint method, the vulnerability probability of gas pipelines is calculated from the perspective of the interaction between disaster hazard intensity and pipeline resistance capacity. It can make up for the subjective deficiency in previous research, which mainly relied on expert experience for evaluation.

(3) Based on the data of karst exploration and field investigation, combined with fuzzy theory, the probability distribution of nodes at different levels of the Bayesian network is calculated step by step, and the hazard probability of disaster events is calculated to be 0.0352. Based on the finite element simulation results, combined with the reliability analysis theory, the vulnerability probability of gas pipelines is calculated to be 0.1038. The disaster-causing possibility of gas pipelines in karst areas is comprehensively measured from two dimensions: the hazard of disaster events and the vulnerability of gas pipelines, and finally it is calculated to be 0.0037.

## Supporting information

S1 File The optimization matrixes of knowledge level, experience level, information source and justice degree. (PDF)

S2 File Expert evaluation results of hazard level. (PDF)

S3 File Random numbers of 8 vulnerable parameters. (XLSX)
