# Supplementary material for: Study on disaster-causing probability evaluation of gas pipeline in karst area
Source: PLoS One. 2025 Feb 3;20(2):e0316820. doi: 10.1371/journal.pone.0316820 (PMC11790121; doi:10.1371/journal.pone.0316820)
Supplement: S1 File — (PDF) [file pone.0316820.s001.pdf]

Table 1 The optimization matrix of knowledge level

	Expert 1	Expert 2	Expert 3	Expert 4	Expert 5	Expert 6
Expert 1	1	1.7321	1.3161	1	5.1966	1
Expert 2	0.5773	1	0.3333	0.1924	0.5773	0.1924
Expert 3	0.7598	3	1	0.7598	1.3161	0.7598
Expert 4	1	5.1966	1.3161	1	1.7321	1
Expert 5	0.1924	1.7321	0.7598	0.5773	1	0.5773
Expert 6	1	5.1966	1.3161	1	1.7321	1

Table 2 The optimization matrix of experience level

	Expert 1	Expert 2	Expert 3	Expert 4	Expert 5	Expert 6
Expert 1	1	0.5773	0.5773	0.5773	0.5773	0.7598
Expert 2	1.7321	1	1	1	1	1.3161
Expert 3	1.7321	1	1	1	1	1.3161
Expert 4	1.7321	1	1	1	1	1.3161
Expert 5	1.7321	1	1	1	1	1.3161
Expert 6	1.3161	0.7598	0.7598	0.7598	0.7598	1

Table 3 The optimization matrix of information source

	Expert 1	Expert 2	Expert 3	Expert 4	Expert 5	Expert 6
Expert 1	1	1.7321	0.7598	1	1.3161	1
Expert 2	0.5773	1	3	1.7321	0.7598	1.7321
Expert 3	1.3161	0.3333	1	1.3161	1.7321	1.3161
Expert 4	1	0.5773	0.7598	1	1.3161	1
Expert 5	0.7598	1.3161	0.5773	0.7598	1	0.7598
Expert 6	1	0.5773	0.7598	1	1.3161	1

Table 4 The optimization matrix of justice degree

	Expert 1	Expert 2	Expert 3	Expert 4	Expert 5	Expert 6
Expert 1	1	1.3161	1	0.7598	1	0.7598
Expert 2	0.7598	1	0.7598	0.5773	0.7598	0.5773
Expert 3	1	1.3161	1	0.7598	1	0.7598
Expert 4	1.3161	1.7321	1.3161	1	1.3161	1
Expert 5	1	1.3161	1	0.7598	1	0.7598
Expert 6	1.3161	1.7321	1.3161	1	1.3161	1
