# Supplementary material for: Study on disaster-causing probability evaluation of gas pipeline in karst area
Source: PLoS One. 2025 Feb 3;20(2):e0316820. doi: 10.1371/journal.pone.0316820 (PMC11790121; doi:10.1371/journal.pone.0316820)
Supplement: S2 File — (PDF) [file pone.0316820.s002.pdf]

Basic event	Expert evaluation results					
	Expert 1	Expert 2	Expert 3	Expert 4	Expert 5	Expert 6
The low level of education (t_1)	L	L	RL	RL	L	RL
The lack of professional skills (t_2)	M	M	RL	RL	M	RL
The weak awareness of pipeline protection (t_3)	RH	RH	RH	RH	M	M
Weak safety awareness (t_4)	RH	RH	RH	H	H	H
Insufficient emergency response capacity (t_5)	RH	RH	H	H	H	H
Pipeline aging (t_6)	RL	RL	RL	L	L	L
Pipeline corrosion (t_7)	H	H	H	H	H	H
The failure of safety facilities (t_8)	RH	RH	RH	RH	RH	RH
Construction technology defect (t_9)	M	M	M	M	RL	RL
The intensity of human activity (t_{10})	RH	RH	RH	RH	H	H
Construction and vibration (t_{11})	RH	RH	RH	RH	M	M
Groundwater extraction (t_{12})	L	L	L	M	L	M
Economic development level (t_{13})	L	L	RL	RL	RL	RL
Legal environment (t_{14})	M	M	H	RH	M	RH
Karst geological development (t_{15})	H					
Groundwater activity (t_{16})	RL					
Overburden characteristics (t_{17})	M					
Structural condition (t_{18})	RH					
Topographic features (t_{19})	M					
Meteorological condition (t_{20})	M	M	RL	M	RL	RH
The lack of safety supervision (t_{21})	RH	RH	RH	H	RH	H
The lack of safety publicity (t_{22})	RL	RL	M	M	M	M
Inadequate emergency support (t_{23})	H	H	H	H	H	H
Unreasonable rules and regulations (t_{24})	RH	RH	RH	H	RH	H
The source of fire (t_{25})	H	H	H	H	H	H
Thermal radiation flux (t_{26})	H	H	H	H	H	H
Smoke and dust (t_{27}),	M	M	M	M	M	RH
Noxious fumes (t_{28})	RH	RH	RH	RH	RH	H
The types of gas leakage (t_{29})	RH	RH	RH	RH	H	H
The scope of fire impact (t_{30})	H	H	H	H	H	H
Radiation time (t_{31})	H	H	H	H	H	H
Distance (t_{32})	H	H	H	H	H	H
The shock wave overpressure (t_{33})	H	H	H	H	H	H
Noise (t_{34})	M	M	M	M	M	RH
Explosion impact (t_{35})	H	H	H	H	H	H
Space-limited situation (t_{36})	M	M	M	RH	H	RH
Human casualties (t_{37})	H	H	H	H	H	H
The destruction of buildings and structures (t_{38})	RH	RH	RH	RH	RH	RH

The destruction of lifeline engineering (t_{39})	M	M	M	RH	RH	RH
The material property loss (t_{40})	M	M	RH	M	M	M
Economic compensation (t_{41})	M	M	RH	M	M	M
Resource waste (t_{42})	RL	RL	RL	M	RL	M
Production and business suspension (t_{43})	M	M	RH	M	M	RH
Atmospheric pollution (t_{44})	RL	RL	RL	M	RL	RH
Soil hardening (t_{45})	RL	RL	RL	M	RL	M
The damage to vegetation (t_{46})	RL	RL	RL	M	M	M
The harm to animals (t_{47})	RL	RL	RL	M	M	M
Early warning capability (t_{48})	H	RH	H	H	H	H
Disaster resistance capability (t_{49})	H	H	H	H	H	H
Disaster relief capacity (t_{50})	H	H	H	H	H	H
Recovery capacity (t_{51})	RH	RH	H	H	H	H
